# Relations among Socioeconomic Status, Perceived Stress, and the Home Language Environment

**DOI:** 10.1017/S0305000923000156

**Published:** 2023-03-14

**Authors:** Emma R. HART, Sonya V. TROLLER-RENFREE, Jessica F. SPERBER, Kimberly G. NOBLE

**Affiliations:** Teachers College, Columbia University, USA

**Keywords:** Perceived Stress, Home Language Environment, Socioeconomic Status, Infant Language Development, LENA

## Abstract

While socioeconomic disparities in the home language environment have been well established, the mechanisms explaining these disparities are poorly understood. One plausible mechanism is heightened stress. The current study investigated whether maternal perceived stress was 1) associated with measures of the home language environment, and 2) mediated the relation between socioeconomic disparities and the home language environment. Data from three independent studies were analyzed, which together comprised 322 mother-child dyads. Two studies included mothers and their six- to twelve-month-old infants (N = 227). The third included mothers and their five- to nine-year-old children (N = 95). Mothers reported their educational attainment, income, and stress. Language Environment Analysis (LENA) measured the home language environment. As has been previously reported, socioeconomic disparities were observed in adult words and conversational turns. Stress did not mediate these associations, nor was it associated with adult words or conversational turns. Alternate mechanisms for future exploration are discussed.

## Introduction

Socioeconomic disparities in language development have been long documented: children from disadvantaged backgrounds tend to score lower on traditional measures of English language skill than do their more advantaged peers (Justice, Jiang, Purtell, Schmeer, Boone, Bates & Salsberry, 2019; Noble, McCandliss & Farah, 2007; Pace, Luo, Hirsh-Pasek & Golinkoff, 2017). The quantity and quality of language that children are exposed to plays an important role in explaining these differences (Brito, 2017; Magnuson, Sexton, Davis-Kean & Huston, 2009; Pace et al., 2017; Perkins, Finegood & Swain, 2013). Indeed, exposure to more words and engagement in reciprocal parent-child conversational interactions are considered key processes for scaffolding language development both in early and middle childhood (Ford, Elmquist, Merbler, Kriese, Will & McConnell, 2020; Merz, Maskus, Melvin, He & Noble, 2020; Weisleder & Fernald, 2013).

In a widely cited study, Hart and Risley (1995) estimated that children raised in disadvantaged socioeconomic circumstances heard up to 30 million fewer words by their third birthday than children growing up in more privileged circumstances. While the validity, magnitude, and interpretation of these findings has been disputed (Dailey & Bergelson, 2022; Golinkoff, Hoff, Rowe, Tamis-LeMonda & Hirsh-Pasek, 2019; Kuchirko, 2019; Sperry, Sperry & Miller, 2019), the essence of this often-cited work has been replicated with added nuance: children from socioeconomically disadvantaged backgrounds do generally tend to hear fewer words at home, *but with notable heterogeneity across families* (Gilkerson, Richards, Warren, Montgomery, Greenwood, Kimbrough Oller, Hansen & Paul, 2017; Rowe, Pan & Ayoub, 2005; Weisleder & Fernald, 2013). Differences in the home language environment have been associated with differences in brain development and, ultimately, language skills (Pierce, Reilly & Nelson, 2020; Romeo, Leonard, Robinson, West, Mackey, Rowe & Gabrieli, 2018; Romeo et al., 2018). Associations between the home environment and language development have been observed as early as in the first two years of life (Halle, Forry, Hair, Perper, Wandner, Wessel & Vick, 2009; Melvin, Brito, Mack, Engelhardt, Fifer, Elliott & Noble, 2017; Noble, Houston, Brito, Bartsch, Kan, Kuperman, Akshoomoff, Amaral, Bloss, Libiger, Schork, Murray, Casey, Chang, Ernst, Frazier, Gruen, Kennedy, Van Zijl, Mostofsky, Kaufmann, Kenet, Anders, Jernigan & Sowell, 2015; Weisleder & Fernald, 2013).

Despite much research on the associations among socioeconomic factors, the home language environment, and child language development, there has been relatively little investigation of the mechanisms that link distal socioeconomic factors, such as parents’ educational attainment or family income, to the verbal interactions they have with and around their children. Some research has suggested that caregiver knowledge is at play (e.g., Rowe, 2008), and indeed, several interventions have focused on improving language-development-related knowledge and skills among caregivers experiencing socioeconomic disadvantage (see Leung, Hernandez & Suskind, 2020; Mendelsohn, Huberman, Berkule, Brockmeyer, Morrow & Dreyer, 2011).

Notably, some scholars have critiqued the assumption that socioeconomic differences in parent-child verbal interactions are primarily driven by deficits in caregiver knowledge and skill (Adair, Colegrove & McManus, 2017; Dudley-Marling & Lucas, 2009; Ellwood-Lowe, Foushee & Srinivasan, 2021; Kuchirko, 2019), and have argued, instead, that the experience of socioeconomic disadvantage may exert structural pressures on caregivers that influence the home language environment in ways unrelated to caregivers’ knowledge or skills (Adair et al., 2017; Dudley-Marling & Lucas, 2009; Ellwood-Lowe et al., 2021; Kuchirko, 2019). Some have also argued that the field should embrace new conceptualizations of optimal language development and home language environments, as traditional assessments of both constructs do not incorporate certain skills (e.g., oral narrative skills) and experiences that are common among children from historically marginalized communities (see Dudley-Marling & Lucas, 2009; Gardner-Neblett, Pungello & Iruka, 2012; Kuchirko, 2019; also see Noble et al., 2021).

It is likely that programs directed towards improving caregiver skills alone may have limited effectiveness if they do not target the underlying structural forces that continuously exert pressures on families. Such structural pressures may lead to increased stress (Algren, Ekholm, Nielsen, Ersbøll, Bak & Andersen, 2018; Glasscock, Andersen, Labriola, Rasmussen & Hansen, 2013; Hackman, Farah & Meaney, 2010; McLoyd, 1998; Senn et al., 2014), which in turn may impact caregivers’ language use with their children (Perkins et al., 2013). Indeed, maternal stress has often been theorized as a critical mechanism driving socioeconomic disparities in child and family outcomes, including caregiver behaviors (see Conger, Wallace, Sun, Simons, McLoyd & Brody, 2002; Evans, Boxhill & Pinkava, 2008; Farah, 2017; Masarik & Conger, 2017; McLoyd, 1998). For example, Family Stress Theory has purported that economic hardship can impose stress and strain on caregivers, affecting how they interact with their children.

Empirical investigation of this theory has suggested that caregivers experiencing greater stress tend to engage in fewer developmentally supportive caregiving behaviors (see Masarik & Conger, 2017 for a review). Less explored, however, is the extent to which stress influences the frequency with which caregivers speak in the home and engage in conversational turns with their children. Some studies have found that higher caregiver stress is associated with lower performance on traditional measures of child language development (D’Souza, Crawford, Buckley, Underwood, Peterson, Bird, Morton & Waldie, 2019; Troller-Renfree, Hart, Sperber, Fox & Noble, 2022), though evidence is mixed (Lehr, Wecksell, Nahum, Neuhaus, Teel, Linares & Diaz, 2016; Lin, Xu, Huang, Jia, Zhang, Yan & Zhang, 2017). It is thus plausible that caregiver stress might influence the home language environment in ways that are important for child language development.

To our knowledge, just one recent study of a small low- to middle-income sample has investigated this, reporting that higher maternal perceived stress and a greater number of stressful life events were associated with fewer words spoken in the home, and fewer parent-child conversational turns (Pierce et al., 2020). The current study aimed to replicate and extend those findings in a larger, socioeconomically diverse group of mothers and their infants and children. This study made use of data from three independent studies, two composed of mothers and their six- to twelve-month-old infants, and the third composed of mothers and their five- to nine-year-old children. Building from previous reports that family socioeconomic status (SES) was associated with measures of the home language environment in two of the samples used in the present study (Brito, Troller-Renfree, Leon-Santos, Isler, Fifer & Noble, 2020; Merz et al., 2020), we hypothesized that higher maternal perceived stress would also be associated with the quality of the home language environment. Specifically, we hypothesized that greater maternal perceived stress would be related to fewer adult words, as well as fewer parent-child conversational turns. Further, we hypothesized that maternal perceived stress would significantly mediate the relation between socioeconomic factors and these measures of the home language environment.

## Methods

### Participants

Data from three separate studies were analyzed, which altogether sampled 322 mother-child dyads. Studies 1 and 2 included mothers and their six- to twelve-month old infants (combined N = 227 dyads, together termed the Combined Infant Sample). Study 3 included mothers and their five-to-nine-year-old children (N = 95 dyads, termed the Child Sample). All three studies recruited mothers from the New York City metropolitan area to study the associations among early experience and child development. Participants were recruited to be intentionally socioeconomically diverse based on maternal educational attainment. Detailed participant demographics for all three samples are presented in Table 1; all three studies included racially and ethnically diverse samples (see Table 1 for details).

Participants from Study 1 included 94 mother-infant dyads recruited from 2016 to 2018. To be included, infants had to be between 5 and 13 months of age, born at or after 36 weeks of gestation, and have no known neurological or developmental complications. All data were collected concurrently, at a single study visit. Participants included 32 six-month-olds, 31 nine-month-olds, and 31 twelve-month-olds (*M* = 9.44 months, *SD* = 2.65). 35% of the children were female.

Participants from Study 2 included 133 mothers recruited from 2019 to 2022 in their third trimester of pregnancy to participate in a longitudinal study of child development from birth to 3 years of age. To be included, pregnant mothers had to be 18 years of age or older, carrying a singleton, at least 35 weeks pregnant, and speak English or Spanish. In addition, the fetus had to be free of known neurological or developmental concerns. The current analyses made use of socioeconomic data collected at the prenatal visit, and perceived stress and home language environment data collected at the 6-month visit (*M* = 7.61 months, *SD* = 1.32). 56% of the children were female.

Participants from Study 3 included 95 mother-child dyads recruited from 2013 to 2018. To be included, children had to be from households that spoke primarily English, born from a singleton pregnancy, born at full-term, and not have any medical or psychiatric problems. Data analyzed in the present paper were collected concurrently at one study visit. Child participants were 5 to 9 years of age (*M* = 7.11 years, *SD* = 1.25). 61% of the children were female.

### Procedure

All protocols were approved by the Institutional Review Board at Teachers College and, for Study 3, Columbia University Medical Center. The data reported here from Studies 1 and 3 were collected from mothers and their children at a single laboratory visit. During this visit, mothers completed surveys to capture information on SES, demographics and perceived stress. They were also invited to take a Language Environment Analysis (LENA) device home, and were provided materials to mail back their LENA device after completion of the recording.

In Study 2, pregnant mothers either visited the research laboratory or research assistants visited their homes during their third trimester, at which time socioeconomic and other demographic information was collected. Mothers and infants subsequently visited the laboratory together when the infant was 6 months of age. During this 6-month visit, mothers were invited to complete a survey on perceived stress, and to take LENA devices home to record the home language environment. Mothers were provided materials to mail their LENA device back to the laboratory after completion of the home recording.

## Measures

### Socioeconomic Status

#### Parental education

Average parental education was calculated by averaging the number of years of education attained by the mother and the second parent. In cases where the reporting mother was the sole parental caregiver, only maternal educational attainment was used.

#### Income-to-Needs

Income-to-needs (ITN) ratios were calculated by dividing participants’ total household income by the poverty threshold for the respective family size for the year of data collection. As expected, ITN values were positively skewed and subsequently log-transformed for use in all analyses. Some participants reported that their income was zero dollars. To enable log transformation, one dollar was added to all income values prior to calculating ITN.

### Perceived Stress

Perceived maternal stress was assessed using the Perceived Stress Scale (PSS-10; Cohen & Williamson, 1988). The 10-item scale assesses the degree to which the respondent has perceived situations as stressful within the last month. Participants responded to each item using a 5-point Likert scale (*0 = never, 4 = very often*). Four items were reverse coded before summing across the items. Higher scores indicated greater perceived stress. Mothers needed to complete at least eight of the ten items for their score to be considered valid. The items showed acceptable reliability in the Combined Infant Sample (*α* = 0.86) and Child Sample (*α* = 0.81).

### Home Language Environment

Language Environment Analysis (LENA) was used to measure the home language environment. The LENA system (LENA Research Foundation, Boulder, CO) is an automated vocalization analysis device that can audio-record the child’s language environment for up to 16 hours. Participants were provided with specially designed child T-shirts to hold the LENA digital language processor (DLP) throughout the recording duration. Strong reliability and validity of the LENA speech identification algorithms has been reported, with over 75% accuracy for both adult and child speech (Gilkerson et al., 2017). LENA has also been validated for Spanish-speakers (Weisleder & Fernald, 2013).

Mothers were provided LENA materials during their visit and instructed to have their child wear the DLP for a full day in the near future when typical caregivers would be present. Once the DLP was returned, the recording was uploaded to a computer and analyzed by the LENA software. The software produces three primary counts: adult words (number of words spoken near the child), conversational turns (number of reciprocal vocalizations by an adult and the target child within 5 seconds), and child vocalizations (defined as a speech segment of any length surrounded by 300 milliseconds or more of non-speech or silence). Adult word counts and adult-child conversational turns were used in the current study.

Additional processing was executed to remove silent periods of 10 minutes or more. This was done to account for the fact that daytime sleep patterns change with developmental age, which could skew the proportion of speech per recording duration. By removing periods of silence of greater than 10 minutes, we ensure that LENA analyses are limited to wakeful time. This process was completed using custom python scripts (see github.com/trollerrenfr/LENA_Scripts). Specifically, segments were removed from the data if there were two consecutive five-minute periods (10 minutes total) in which: 1) there was noise or silence for more than 180 seconds, 2) there were no adult words detected, 3) there were no conversational turns detected, and 4) there were fewer than 10 child vocalizations. Following the removal of data segments that met these criteria, LENA counts were then divided by the duration of LENA recording, to create average hourly counts of adult words and conversational turns. Recordings less than two hours in duration were set as missing in analyses.

Although LENA software has been formally validated for reliable use with children up to four years of age, it has also been used with older children (Romeo et al., 2018; Vohr, Topol, Watson, St Pierre & Tucker, 2014; Wang, Pan, Miller & Cortina, 2014). Previous work tested the reliability of the LENA software at older ages within the Child Sample, reporting a high correlation (*r* = .74, *p* < .001) between estimates of child vocalizations using LENA software vs. hand-coded transcriptions (see Merz et al., 2020 for details). This correlation is in line with estimations of the LENA software accuracy among younger children (76%; Gilkerson et al., 2017).

### Analytic Samples

Given similarity in recruitment methods and maternal demographics for Studies 1 and 2, the samples were combined to provide greater analytic power. Together, Studies 1 and 2 formed the Combined Infant Sample. Study 3 comprised the Child Sample. See Table 1 for details on the similarities and differences between Studies 1 and 2, and between the Combined Infant Sample and Child Sample. A dummy variable for Study was included in all Combined Infant Sample analyses. Several sensitivity checks were also performed to ensure that the combination of these samples did not bias the results (see online supplemental file).

As presented in Table 1, Welch’s *t*-tests showed relatively few statistically detectable differences between the samples.^1^ Perceived stress scores were, on average, lower in Study 2 (*M* = 11.44, *SD* = 6.92) than Study 1 (*M* = 14.13, *SD* = 6.45; *t* = 2.84, *p* = .005). Notably, average stress levels in Study 1 just barely fell within the ‘moderate stress’ range (i.e., between 14–26), while average stress in Study 2 fell within the ‘low stress’ range (Nedea, 2020). Average stress in the Child Sample, however, was squarely within the ‘moderate stress’ range (*M* = 17.60, *SD* = 6.19), and higher than the Combined Infant Sample (*M* = 12.57, *SD* = 6.84, *t* = −6.24, *p* < .001). Families in the Combined Infant Sample (*M* = 5.10, *SD* = 6.70) had higher ITN ratios than families in the Child Sample (*M* = 2.59, *SD* = 2.45; *t* = 4.83, *p* < .001). As might be expected developmentally, children in the Child Sample heard fewer adult words per hour (*t* = 2.63, *p* = .009), but engaged in more conversational turns per hour (*t* = −4.12, *p* < .001) than those in the Combined Infant Sample.

### Outliers

Outliers in LENA data were investigated, with particular attention toward low-duration recordings. First, to ensure that low-duration recordings did not skew the data, all LENA recordings less than 2 hours in duration were set as missing. There were 5 participants with short LENA recording durations in the Combined Infant Sample, and 3 participants in the Child Sample. Next, all LENA data was set as missing for participants with adult word counts or conversational turn counts greater than three standard deviations from the respective sample average. There was one participant with outlier LENA data in the Combined Infant Sample, and none in the Child Sample. The participant with outlier data in the Combined Infant Sample had LENA recordings that were less than five hours in duration. Finally, in the Child Sample, one additional participant’s LENA values were set to missing, due to parent report of incorrect LENA device usage (parent carried the device in their pocket).

Additional outliers were addressed for continuous non-standardized measures (i.e., average educational attainment, ITN ratios). In the Combined Infant Sample there were four participants with ITN ratios greater than three standard deviations from the mean (ITN > 31.18), which were winsorized to be exactly 3 standard deviations from the mean (31.18). In the Child Sample there were two families with income-to-needs ratios greater than three standard deviations from the average (ITN > 11) which were winsorized to be 11. There were no outliers in average educational attainment in either sample.

### Missing Data

Prior to formal missing data imputation procedures, we estimated missing income values using available data to address cases of income missingness. Indeed, in many cases families did not provide precise income figures due to lack of comfort in sharing this information. In Study 1, there were 3 participants who did not report their income, but did indicate which “bin” their income fell within. For these participants, income was assigned to be the median value of the bin they reported (e.g., if bin was “$10,000 to $15,000”, then their income level was assigned as $12,500). In Study 2, 17 participants did not provide income data at the prenatal visit. However, 11 of them subsequently provided income information at the 12-month visit. In these cases, the 12-month income information was used for analyses. In Study 3, there were 4 participants who did not report their income, but who did indicate their income bin. As in Study 1, the average of their indicated bin was used as their income for analyses.

After estimating income for these cases, there was full data coverage for 54.2% of the Combined Infant Sample (134 completed LENA, 203 completed PSS, 216 reported ITN, 227 reported parental educational attainment), and 76.8% of the Child Sample (77 participants completed LENA, 91 completed PSS, 95 completed ITN, and 95 reported parental educational attainment). Full Information Likelihood Estimation (FIML) was utilized to account for missingness (see supplemental file for more details on missingness and for analyses performed without the use of FIML; we note that primary findings were consistent across models that did and did not use FIML.).

### Analytic Plan

All analyses were executed using Stata 16.0 (StataCorp, 2019). Primary analyses were performed separately for the Combined Infant Sample and the Child Sample. For both samples, linear regression analyses were performed to identify whether perceived stress was associated with the home language environment (adult words and conversational turns) when controlling for educational attainment and ITN. In each model, perceived stress, average educational attainment and ITN were entered as independent variables. Home language environment measures (average hourly adult word count and average hourly conversational turn count) were entered as dependent variables. Models also included controls for child age at the time of LENA recording, and LENA recording duration. In addition, study affiliation (Study 1 versus Study 2) was entered as a covariate for the Combined Infant Sample models. Next, mediation analyses were performed to determine whether maternal perceived stress mediated any of the statistically significant relations between the socioeconomic factors and home language environment measures. In these analyses, perceived stress served as the mediator, and either adult word count or conversational turn count served as the dependent variable. The same covariates were included in these models as were included in the regressions. All variables were standardized prior to analyses, and standardized Betas were reported (unless otherwise noted).

Several additional sensitivity checks were performed to further test the robustness of findings. These tests are detailed and reported in the online supplementary file. Across models, we tested whether the substantive findings from our primary models held. For both the Combined Infant Sample and Child Sample, these included the introduction of additional covariates beyond those included in the primary models for child sex, maternal age, race (i.e., reference group was whether the parent was White, with dummy variables for whether the parent was Black or whether the parent reported a different racial identity), whether the parent was Hispanic, and duration of time between maternal reports of perceived stress and recording of the home language environment (given that there was often a lag between when mothers completed the PSS and their home LENA recordings). These controls were chosen for both theoretical reasons (i.e., differences in the lived realities of families from different races due to racism and other structural inequities) and empirical reasons (i.e., associations with both independent and dependent variables).

As a final series of robustness checks, we tested the relation among the home language environment and alternate measures of stress for which we had poorer data coverage. Exploratory regression models were executed to test the relations among the home language environment measures and two additional measures of stress: 1) maternal hair cortisol concentration and 2) a stress composite composed of stress-related measures. These results, and measurement details, are presented in the online supplementary file.

## Results

### Descriptive Statistics

The bottom, left-hand side of Table 2 details the correlations between the key variables of interest within the Combined Infant Sample. Perceived stress was not associated with adult word count or conversational turn count. Perceived stress was also not associated with ITN or educational attainment. Both greater ITN and greater education were moderately associated with greater adult word count and greater conversational turn count.

The top, right-hand side of Table 2 presents the correlations between the key variables of interest within the Child Sample. As was the case for the Combined Infant Sample, perceived stress was not associated with either measure of the home environment or either measure of SES. In this sample, ITN was not statistically significantly associated with adult word count of conversational turn count, but educational attainment showed moderate to large correlations with both of these outcomes.

### Perceived Stress, SES, and Adult Words

First, regression analyses were performed to determine the unique associations between perceived stress and adult word count. Table 3 presents these results for both samples. In both the Combined Infant Sample (*β* = −.01, *SE* = .08, *p* = .89) and Child Sample (*β* = .02, *SE* = .10, *p* = .84), there was no association between perceived stress and adult word count. When controlling for ITN, parental educational attainment demonstrated a moderate association with adult words in the Combined Infant Sample (*β* = .32, SE = .08, *p* < .001) and in the Child Sample (*β* = .49, *SE* = .14, *p* = .001). The coefficients for ITN were smaller and statistically non-significant in the Combined Infant Sample (*β* = .13, *SE* = .10, *p* = .20) and the Child Sample (*β* = −.16, *SE* = .15, *p* = .29).

### Perceived Stress, SES, and Conversational Turns

Subsequent regression analyses were executed to test whether perceived stress was related to conversational turn counts (see Table 3). As was the case for adult word count, perceived stress was neither associated with conversational turn count in the Combined Infant Sample (*β* = .02, *SE* = .09, *p* = .84), nor in the Child Sample (*β* = .05, *SE* = .11, *p* = .63). As was also the case for adult word count, parental educational attainment showed moderate associations with conversational turns in both the Combined Infant Sample (*β* = .27, *SE* = .09, *p* = .002) and the Child Sample (*β* = .31, *SE* = .14, *p* = .03). ITN did not statistically significantly relate to conversational turns in either the Combined Infant Sample (*β* = .13, *SE* = .11, *p* = .23) or Child Sample (*β* = .02, *SE* = .15, *p* = .88).

### Mediation Models

A mediational analysis was conducted to explore whether there was an indirect effect of parental educational attainment on home language inputs through perceived stress, since education was related to both adult word count and conversational turn count in both samples. There was no indirect effect of educational attainment on adult word counts (Combined Infant Sample: *β* = .00, *SE* = .00, *p* = .81; Child Sample: *β* = .00, *SE* = .01, *p* = .79) or conversational turns (Combined Infant Sample: *β* = .00, *SE* = .00, *p* = .95; Child Sample: *β* = −.01, *SE* = .01, *p* = .67) through stress.

### Robustness Checks

Several robustness checks were performed to test the sensitivity of these results. Analytic details are provided in the online supplementary file (see Tables S1–S7). These analyses included models that: considered Study 1 and 2 independently; did not use FIML; contained additional control variables; and included a restricted sample of only five- to seven-month-old infants from Studies 1 and 2. Across all robustness checks, perceived stress was not associated with measures of the home language environment. The relations between the socioeconomic variables and home language outcomes were more variable depending on model specification. Taken together, parental educational attainment appeared to be more consistently statistically significantly related to home language measures, though the magnitude of the coefficients was fairly variable from one model to the next.

Additional exploratory analyses were performed with limited sample sizes to investigate whether physiological stress, measured via maternal hair cortisol concentration, and Stress Composites, composed of several stress-related measures, were associated with differences in the home language environment (See Table S8 and S9, respectively). We found that neither hair cortisol concentration nor the Stress Composites were associated with hourly adult word count or conversational turn count.

## Discussion

While previous work has investigated socioeconomic disparities in the home language environment, there has been less attention directed towards understanding the mechanisms that explain these disparities. The present study tested whether maternal perceived stress directly related to, and accounted for socioeconomic differences in, the home language environments of six- to twelve-month-old infants and five- to nine-year-old children. While our results replicated past work showing some socioeconomic differences in home language inputs, we found no evidence that perceived stress explained these differences. Supplemental analyses suggested the same pattern of findings for maternal physiological stress and a composite of several stress-related measures. Together, these findings suggest the possibility that perceived stress does not influence the home language environment. These findings highlight the importance of future work investigating which factors, if not perceived stress, drive socioeconomic differences in the home language environment.

These results contradict findings reported by Pierce et al. (2020), which was the first study, to our knowledge, that investigated these relations. Pierce et al. (2020) found that greater perceived stress was associated with lower home language environment counts in families with infants. Indeed, our Combined Infant Sample focused on infants of the same age, used similar methodologies, and reported similar perceived stress levels as the Pierce et al. study. Of note, the two did differ on some sample characteristics which could potentially explain differences in study findings. Pierce and colleagues’ findings were observed among a small sample (n = 22) of primarily low- and middle-income families, whereas our Combined Infant Sample spanned a larger socioeconomic range (including high-income families). Additionally, LENA counts were generally higher for participants in our sample than Pierce’s sample. Our sample was also composed of mothers who were, on average, older than mothers in Pierce and colleagues’ sample. Additional replications are needed to further probe the links between perceived stress and the home language environment, and to better understand which characteristics moderate these findings, especially given the relatively large standard errors associated with our estimates.

Beyond testing these direct associations between stress and the home language environment, our study also aimed to test whether stress mediated the relation between parental educational attainment and the home language environment. We found no consistent evidence that socioeconomic factors were statistically significantly correlated with perceived stress or, in our supplemental analyses, with physiological stress. Importantly, we observed this pattern in two samples with different average perceived stress levels (mothers in the Child Sample reported ‘moderate stress’, while mothers in the Combined Infant Sample reported ‘low stress’; Nedea, 2020). While some previous research has also reported no relation between these constructs (e.g., Ursache, Noble & Blair, 2015), other studies have found that lower SES is generally associated with higher perceived stress (Algren et al., 2018; Glasscock et al., 2013; Senn, Walsh & Carey, 2014). Importantly, such heterogeneity in findings could be due to the fact that the circumstances and experiences associated with low income and low educational attainment vary widely across families (DeJoseph, Sifre, Raver, Blair & Berry, 2021). As such, socioeconomic disadvantage may be related to perceived stress in some cases, but not others. Interestingly, ITN and educational attainment showed small to moderate associations (generally statistically significant) with a composite of various stress-related measures in our supplemental models.

It is important to consider that perceptions of stress are distinct from the experience of stressors themselves (see Troller-Renfree et al., 2022 for review). While socioeconomic disadvantage may be associated with exposure to more stressors (Hackman et al., 2010; McLoyd, 1998), perceptions of stress may be largely independent from the number of stressors a family experiences, and may, instead, be driven by individual, trait-level variability in processing and mental health. Individuals experiencing socioeconomic disadvantage and associated stressors may also adapt so that they are better equipped to handle this stress and, as such, perceive their day-to-day stress levels as relatively low (Ellis, Abrams, Masten, Sternberg, Tottenham & Frankenhuis, 2020). Importantly, measures of perceived stress, such as the PSS, may be limited in their ability to capture the nuances of stress appraisal across contexts where stress is more or less chronic and/or severe. It will be worthwhile for future studies to test whether other forms of stress, or stressors themselves, relate to the home language environment. Although we found no associations between the home language environment and alternate measures of stress – including maternal hair cortisol concentration and a composite composed of multiple stress-related measures – these analyses were limited by small sample size. Future work should investigate these relations in larger samples.

In line with past work documenting socioeconomic differences in home language inputs (Gilkerson et al., 2017; Rowe et al., 2005; Weisleder & Fernald, 2013), we generally found that socioeconomic factors were associated with higher hourly counts of adult words and conversational turns (see Brito et al., 2020; Merz et al., 2020 for original reporting of these results for Studies 1 and 3). However, in the models reported here, there appeared to be more consistent evidence for links between the home language environment and parental educational attainment rather than family income-to-needs ratios. Notably, our supplemental models showed variability in these associations across different model specifications in terms of both coefficient magnitude and statistical significance. Contrary to our hypothesis, we did not find evidence that perceived stress mediated these links. These results raise questions about which factors *do* explain socioeconomic differences in the home language environment.

New insight may be gleaned from future work that moves beyond investigations of individual-level factors (i.e., perceived stress) to focus, instead, on larger environmental factors that may drive these relations. For example, the experience of various structural inequities associated with socioeconomic disadvantage – such as income inequality, housing and food insecurity, unemployment, lack of access to child care, and racism and discrimination – could explain socioeconomic differences in the home language environment. Such structural inequalities may influence caregivers’ home language behaviors through mechanisms that are unrelated to either caregiver competencies or caregivers’ reported perceptions of stress. Indeed, experimentally induced financial scarcity has been shown to decrease child-directed speech even among middle- to high-income individuals (Ellwood-Lowe et al., 2021). Consideration of the influence of such larger forces may prove valuable in understanding why SES is associated with the home language environment, and in imagining potential interventions to address these factors. If structural factors play an important role in explaining socioeconomic differences in early language development, then interventions directed towards improving caregiver knowledge and stress alone may fail without changes to these larger environmental conditions.

### Limitations

The current study was limited by its use of mostly concurrent data. Future longitudinal investigation may yield new insight about the relations of perceived stress, socioeconomic circumstances, and the home language environment as children develop. Indeed, it is possible that more cumulative measures of stress collected longitudinally will relate differently to measures of the home language environment than measures that capture relatively recent stress, as used in the current study. The data were also limited by the use of home language measures that captured general speech in the home, and not child-directed speech specifically (see Dailey & Bergelson, 2022). It is possible that measures of maternal child-directed speech are affected by stress in ways that the larger home language environment is not. Future work should explore this possibility. The current study was both strengthened and limited by its use of data from three separate samples. While we consistently found no evidence of a relation between maternal perceptions of stress and the home language environment across these samples, we encourage future investigation of these associations in other, even larger, samples. Indeed, it is possible that unobservable differences between these samples contributed bias to the results, although the inclusion of a control for study affiliation, and execution of a variety of sensitivity checks, reduces this possibility. While the current study used a larger sample than past work, the relatively large standard errors on our estimates suggest that future analyses would benefit from investigation of these relations in still larger samples which are powered to estimate associations between stress and the home language environment with increased precision. Finally, as previously noted, future work will benefit from consideration of other theoretically-motivated factors that may explain socioeconomic differences in the home language environment.

## Supplementary Material

supplemental file

## Figures and Tables

**Table 1.** Descriptives for Studies 1, 2, and 3

	Combined Infant Sample							Child Sample			
		
	Study 1			Study 2				Study 3			
					
	Mean / %	Min	Max	Mean / %	Min	Max	Study 1 vs. Study 2	Mean / %	Min	Max	Child vs. Combined Infant

Parent Age	31.72 (5.93)	20.00	47.00	31.74 (5.69)	19.00	44.00	ns	34.68 (6.57)	23.00	51.00	***

Child Age	9.44 (2.65)	5.72	15.12	7.61 (1.32)	5.36	11.51	***	85.29 (14.97)	62.27	119.64	***

Female Child	35%			56%			**	61%			*

Socioeconomic Status											

ITN Ratio	4.10 (4.68)	0.06	24.74	5.81 (7.74)	0.00	31.18	*	2.59 (2.45)	0.17	11.00	***

Education	14.93 (3.74)	6.00	22.00	14.88 (3.23)	7.00	22.00	ns	14.14 (2.63)	6.50	20.00	*

Ethnicity											

Hispanic/ Latino	35%			50%			*	46%			ns

Not Hispanic/ Latino	51%			50%			ns	54%			ns

Prefer Not to Answer	14%			0%			***	0%			***

Race											

A.I./ N.A.	3%			3%			ns	1%			ns

Asian	3%			8%			ns	0%			***

Black	18%			20%			ns	39%			***

N.H./P.I.	1%			0%			ns	0%			ns

White	31%			37%			ns	19%			**

“Other”	21%			29%			ns	40%			*

Prefer Not to Answer	22%			4%			***	0%			***

LENA Recording											

Recording Duration	9.35 (2.56)	2.66	13.90	9.82 (2.74)	4.72	15.83	ns	13.59 (3.30)	4.41	16.00	***

Adult Words	1431.19 (754.15)	70.78	3410.66	1483.22 (774.48)	379.69	3618.44	ns	1211.85 (576.95)	169.75	2621.62	**

Conversational Turns	33.76 (17.51)	3.17	89.00	34.44 (18.09)	7.67	85.72	ns	48.41 (27.39)	0.45	132.26	***

Perceived Stress	14.13 (6.45)	0.00	28.00	11.44 (6.92)	0.00	35.00	**	17.60 (6.19)	4.00	31.00	***

* p < 0.05

** p < .01

*** p < .001

+ p < .10

*Note.* Study 1 included 94 participants. Study 2 included 133 participants. Study 3 included 95 participants. Maternal age is reported in years. Child age is reported in months. Education reflects average years of parental educational attainment. “A.I.” indicates “American Indian,” “N.A.” indicates “Native Alaskan,” “N.H.” indicates “Native Hawaiian,” “P.I.” indicates “Pacific Islander.” LENA recording duration is reported in hours. Adult word, conversational turn, and infant vocalization counts reflect average hourly counts. Percentages for racial group are rounded. Significance stars reported in the “Study 1 vs. Study 2” column reflect statistically significant differences in the characteristics of Study 1 and Study 2 based on Welch’s t-tests. Significance stars reported in the “Child vs. Combined Infant” column reflect statistically significant differences between characteristics of the Combined Infant Sample and Child Sample based on Welch’s t-tests.

**Table 2.** Correlation Matrix- All Studies

	1	2	3	4	5	6
(1) Perceived Stress	–	−0.01	0.03	−0.10	−0.14	−0.10
(2) Adult Words	−0.03	–	0.78***	0.18	0.38***	−0.07
(3) Conversational Turns	0.03	0.74***	–	0.15	0.24*	0.04
(4) Income-to-Needs	−0.03	0.25**	0.22*	–	0.68***	−0.02
(5) Education	0.02	0.39***	0.31***	0.39***	–	0.06
(6) Child Age	0.17+	−0.11	0.06	0.05	−0.04	–

Note.

+ *p* < .10

* *p* < 0.05

** *p* < .01

*** *p* < .001

*Note.* The bottom, left-hand side of the matrix presents correlations for the Combined Infant Sample (Study 1 and Study 2). The top, right-hand side of the matrix presents correlations for the Child Sample (Study 3). Home language environment measures reflect hourly rates with silent periods removed. Income-to-needs-ratios were log-transformed.

**Table 3.** Associations Between Stress and Home Language Environment

	Combined Infant Sample				Child Sample			
	
	Adult Word Count		Conversational Turn Count		Adult Word Count		Conversational Turn Count	
			
	*β*	*SE*	*β*	*SE*	*β*	*SE*	*β*	*SE*

Perceived Stress	−0.01	0.08	0.02	0.09	0.02	0.10	0.05	0.11

Education	0.32***	0.08	0.27**	0.09	0.49**	0.14	0.31*	0.14

Income-to-Needs	0.13	0.10	0.13	0.11	−0.16	0.15	0.02	0.15

Covariate Set #1	yes		yes		yes		yes	

FIML used?	yes		yes		yes		yes	

Observations	227		227		95		95	

Note.

+ *p* < .10

* *p* < 0.05

** *p* < .01

*** *p* < .001

*Note.* The Combined Infant Sample was composed of infants from Studies 1 and 2. The Child Sample was composed of participants from Study 3. Home language environment measures reflect hourly rates with silent periods removed. Covariate Set #1 included: LENA recording duration and child age at time of recording. Study affiliation was also included as a covariate in the Combined Infant Sample analyses. All analyses were performed using FIML. Income-to-needs-ratios were log-transformed.

## Footnotes

1 We tested for statistical differences in the key variables after the removal of outliers (see following section for details on outlier removal).
