# Supplementary material for: Relations among Socioeconomic Status, Perceived Stress, and the Home Language Environment
Source: J Child Lang. Author manuscript; Available in PMC 2024 Sep 14. (PMC10500036; doi:10.1017/S0305000923000156)
Supplement: supplemental file [file NIHMS1913178-supplement-supplemental_file.pdf]

Supplemental Materials for

Relations among Socioeconomic Status, Perceived Stress, and the Home Language Environment

Additional Details on Missing Data

We tested the correlations between LENA missingness on the basis of our independent variable of interest, perceived stress, as well as educational attainment and ITN. We found that there were generally small, negative associations between missingness and the measures of socioeconomic status. These were statistically significant in the Combined Infant Sample (ITN: $r = -.15$, educational attainment: $r = -.17$). Missingness was not associated with perceived stress scores. We proceeded in using FIML to contend with missing data. As a robustness check, we also executed all core models without FIML, to ensure that the use of FIML did not bias the results.

Robustness Checks

Analytic Plan

A series of additional analyses were executed to test the sensitivity of the primary analyses. We were interested to see whether the main findings (that perceived stress was not associated with measures of the home language environment) held true regardless of modeling approach. Tables S1 and S2 present the primary models in two new ways: 1) without FIML and with the inclusion of additional control variables, 2) with FIML and with these additional controls. These analyses were performed to test the sensitivity of the primary findings to the use of FIML and additional covariates.

Subsequent follow-up analyses were performed using either Study 1 or 2, instead of the full Combined Infant Sample, to test whether the primary findings were consistent when these studies were analyzed separately. Given smaller sample sizes, for which the models did not converge with the use of FIML, these analyses were performed without FIML. Table S3 includes

analyses for only participants included in Study 1 with the primary set of controls and additional controls, and Table S4 presents these same analyses for just the participants included in Study 2. Finally, Table S5 presents findings from an analysis performed with only five- to seven-month-olds from the full Combined Infant sample. This analysis was used to test whether the primary analyses were consistent for families with infants falling within this narrower age range given that the Combined Infant Sample otherwise included infants who together comprised a broader age range.

Results

Analyses were performed to test whether the primary results held true with the inclusion of additional controls and without the use of FIML. Across all models, there were very small, statistically non-significant associations between stress and the measures of the home language environment.

Physiological Stress and the Home Language Environment

Exploratory analyses were conducted using participants from Study 1 (six- to nine-month olds) and Study 3 (five- to nine-year olds) to test whether a measure of physiological stress, hair cortisol concentration, was associated with characteristics of the home language environment.

Measures

Physiological Stress

Maternal hair samples were collected to index cortisol deposited over the past three months. A research staff member cut a small section of hair proximal to the posterior vertex of the mother’s scalp. Each hair sample weighed at least 15 mg and was trimmed to be approximately 3 cm long (measured from the end closest to the root), thereby containing cortisol

deposited during roughly the past three months. Samples were stored at −40 degrees Celsius until they were sent to the University of Massachusetts for analysis. Samples were processed and analyzed using previously validated methods (see Davenport et al., 2006; Meyer et al., 2014) Briefly, each sample was weighed, washed twice in isopropanol to remove external contaminants, ground to a fine powder, and extracted with methanol. The methanol extract was evaporated, re-dissolved in assay buffer, and analyzed in duplicate along with standards and quality controls by a sensitive and specific enzyme-linked immunosorbent assay (Salimetrics, Carlsbad, CA). Assay readout was converted to pg cortisol per mg dry hair weight (pg/mg). Intra- and inter-assay coefficients of variation for this assay were <10%. Hair cortisol values were natural log-transformed to correct for skew, similar to previous methods (see Chen et al., 2016). Parents who indicated that they had used steroid medications in the past three months were excluded from analysis (Study 1: n = 9; Study 3: n = 13). There were no significant associations between hair cortisol and potential confounds, including hair washing frequency, use of oral contraceptives, and use of hair dye.

Analytic Plan

Before log-transforming the hair cortisol values, we investigated outliers in the data. In Study 1, there was one participant with a hair cortisol concentration greater than three standard deviations from the Study 1 sample mean (greater than a value of '83'). This value was winsorized to the next viable value, '74.30'. In Study 3, there were two participants with concentrations above 1,200. These participants' data was set to missing out of concern about the biological implausibility of these samples. Then, we assessed whether there were any additional outliers. There was one participant with a concentration greater than three standard deviations from the Study 3 sample mean (greater than a value of '261'). Visual inspection confirmed that

the value was an outlier. This participant’s data was winsorized to the next viable value (‘154’). In total, 62 mothers from Study 1 provided usable hair cortisol data, and 63 mothers from Study 3 provided usable data. Both LENA data and hair cortisol data were only available for 47 participants from Study 1 and 50 participants from Study 3. Regression analyses were executed using the approach described in the main manuscript to test whether physiological stress was associated with average hourly adult word count and conversational turn count. Log-transformed hair cortisol data was used in all analyses.

Results

Results can be found in Table S6. Of note, in Study 1, hair cortisol was not statistically significantly correlated with perceived stress (r = .17, p = .21), ITN (r = -.09, p = .51), educational attainment (r = -.13, p = .32), adult word count (r = -.17, p = .25), or conversational turn count (r = -.01, p = .97). In Study 3, the pattern was similar: hair cortisol was not statistically significantly correlated with perceived stress (r = .02, p = .89), ITN (r = -.11, p = .41), educational attainment (r = -.17, p = .18), adult word count (r = -.08, p = .56), or conversational turn count (r = -.11, p = .44). The analyses suggested that there was no relation between physiological stress and adult word count nor between physiological stress and conversational turn count in either Study 1 or Study 3. Notably, these analyses were executed with small samples (n = 47, 50) and, as such, should be interpreted with caution.

Stress Composite and the Home Language Environment

Exploratory analyses were also conducted to examine whether Stress Composites, comprised of all of the available stress-related measures for each study, were associated with home language environment outcomes. The main manuscript limited analyses to the Perceived

Stress Scale, as it was (1) collected in all three studies, (2) is a widely used measure of stress, and (3) was measured concurrently with the home language environment. While the measures below varied somewhat in their availability across studies, and not all were collected concurrently with home language environment data (see below), for the sake of completeness we constructed composite measures employing all stress measures available in a given study. As described below, results were unchanged from the main analyses using only the Perceived Stress Scale.

Measures

Perceived Stress. Perceived maternal stress was assessed using the Perceived Stress Scale (PSS-10; Cohen & Williamson, 1988). The 10-item scale assesses the degree to which the respondent has perceived situations as stressful within the last month. Participants responded to each item using a 5-point Likert scale (*1 = never, 5 = very often*). Four items were positively started and, thus, reverse coded before summing across the items. Higher scores indicated greater perceived stress. Mothers needed to complete at least eight of the ten items for their score to be considered valid. The Perceived Stress Scale was measured in all three studies, concurrently with the home language environment assessment. However, the scale was also collected prenatally in Study 2, approximately 6 months prior to the measurement of the home language environment. For consistency across the Study 2 stress measures used in this analysis, the prenatal measure of the Perceived Stress Scale was used for Study 2. The items showed good reliability (α =.87, .79, .82 for studies 1, 2, and 3, respectively).

Household Chaos. Household chaos was measured through the Confusion, Hubbub, and Order Scale (CHAOS; Matheny et al., 1995). The CHAOS is a 15-item survey designed to measure the order, routine, and disorganization of the home environment. Participants responded to each item as “true of their home” more or less than half of the time. Before summing the

items, positively stated items were reverse scored. Higher scores indicated greater household chaos. Mothers needed to answer at least 12 of the 15 items to have a valid CHAOS score. Household Chaos was measured in Study 1 concurrently with the home language environment assessment. In Study 2, Household Chaos was measured prenatally, approximately 6 months prior to measurement of the home language environment. Household Chaos was not measured in Study 3. The items demonstrated acceptable reliability (α =.83, .73, for studies 1 and 2, respectively).

Negative Life Experiences. Negative life experiences were measured using the Life Experiences Survey (LES; Sarason et al., 1978). Participants indicated whether they had experienced 44 life events over the course of the past year (e.g., divorce, death). For each event they experienced, mothers rated the impact of the event on their life using a 7-point Likert scale ranging from negative 3 (extremely negative impact) to positive 3 (extremely positive impact). Scores from negatively-rated events were reverse scored and summed to create a total negative events impact score. Mothers needed to respond to at least 80% of the items to be included in analyses. This measure was collected in all three studies. In Study 1 and 3, Negative Life Experiences were measured concurrently with the home language environment assessment. Of note, the Life Experiences Survey only included 43 items in Study 3. In Study 2, Negative Life Experiences were measured prenatally, approximately 6 months prior to measurement of the home language environment. The items demonstrated acceptable reliability (α = .67, .63, for studies 1 and 2, respectively).

Material Deprivation. Material deprivation was assessed using the Material Deprivation Scale (Pilkauskas et al., 2012). The 14-item questionnaire asks participants if they have experienced material hardships in the past year (e.g., paying bills, providing food for their

family, affording medical care). For each item, participants responded whether they had or had not experienced the hardship. The number of hardships experienced was tallied to create a total score, with higher scores indicating greater material hardship. Mothers needed to answer at least 12 of the 14 questions to be included in analyses. Material Deprivation was measured in all three studies. In Studies 1 and 3, Material Deprivation was measured concurrently with the home language environment assessment. In Study 2, Material Deprivation was measured prenatally, approximately 6 months prior to measurement of the home language environment. The items showed good reliability (α =.72, .78, .67, for studies 1, 2, and 3, respectively)

Analytic Plan

Regression analyses were executed together for the Combined Infant Sample as well as separately for Studies 1-3 given several differences in the collection of stress-related measures, as described above. Stress Composites were formed by standardizing and averaging the available stress measures for each study. Composites were considered valid for a participant if they included at least two measures of stress. Regression analyses were performed using education, ITN, and the full set of covariates.

Results

Results can be found in Table S7. In Study 1, the Stress Composite was statistically significantly correlated with ITN (r = -.34, p = .001), was marginally significantly correlated with educational attainment (r = -.20, p = .07), and adult word count (r = -.23, p = .06), but not conversational turn count (r = -.12, p = .34). In Study 2, the Stress Composite was marginally statistically significantly correlated with ITN (r = -.15, p = .08) and statistically significantly correlated with educational attainment (r = -.24, p = .006), but not statistically significantly correlated with adult word count (r = -.16, p = .20) or conversational turn count (r = -.10, p =

.42). Finally, in Study 3, the Stress Composite was statistically significantly associated with ITN (r = -.23, p = .03), and educational attainment (r = .31, p = .002), but not adult word count (r = -.15, p = .19) or conversational turn count (r = -.12, p = .31). The regression models, together, suggested that the Stress Composites were not associated with measures of the home language environment, in line with all of the aforementioned models.

References

Chen, R., Muetzel, R. L., El Marroun, H., Noppe, G., van Rossum, E. F. C., Jaddoe, V. W., … Tiemeier, H. (2016). No association between hair cortisol or cortisone and brain morphology in children. *Psychoneuroendocrinology, 74*, 101–110. https://doi.org/10.1016/J. PSYNEUEN.2016.08.023

Cohen, S., & Williamson, G. (1988). Perceived stress in a probability sample of the United States. In S. Spacapan & S. Oskamp (Eds.), *The social psychology of health.* Newbury Park, CA: Sage Publishers.

Davenport, M. D., Tiefenbacher, S., Lutz, C. K., Novak, M. A., & Meyer, J. S. (2006). Analysis of endogenous cortisol concentrations in the hair of rhesus macaques. *General and Comparative Endocrinology, 147*(3), 255–261. https://doi.org/10.1016/J.YGCEN.2006.01.005

Matheny Jr, A. P., Wachs, T. D., Ludwig, J. L., & Phillips, K. (1995). Bringing order out of chaos: Psychometric characteristics of the confusion, hubbub, and order scale. *Journal of applied developmental psychology*, *16*(3), 429-444.

Meyer, J., Novak, M., Hamel, A., & Rosenberg, K. (2014). Extraction and analysis of cortisol from human and monkey hair. *Journal of Visualized Experiments: JOVE,* (83), e50882. https://doi.org/10.3791/50882

Pilkauskas, N. V., Currie, J. M., & Garfinkel, I. (2012). The great recession, public transfers, and material hardship. *Social Service Review*, *86*(3), 401-427.

Sarason, I. G., Johnson, J. H., Siegel, J. M. (1978). Assessing the impact of life changes: Development of the Life Experiences Survey. *Journal of Consulting and Clinical Psychology, 46*(5), 932-946.

Table S1

Relations among SES, Stress, and LENA- Combined Infant Sample Robustness Checks

	Adult Word Count				Conversation Turn Count			
	Model 1		Model 2		Model 3		Model 4	
	β	*SE*	*β*	*SE*	*β*	*SE*	*β*	*SE*
Perceived Stress	0.01	0.09	0.00	0.08	0.03	0.09	0.02	0.08
Education	0.20+	0.11	0.19+	0.10	0.10	0.11	0.13	0.10
Income-to-Needs	0.12	0.11	0.11	0.10	0.11	0.11	0.10	0.11
Covariate Set #1	yes		yes		yes		yes	
Covariate Set #2	yes		yes		yes		yes	
FIML used?	no		yes		no		yes	
Observations	123		227		123		227	

Note. + $p < .10$, * $p < 0.05$, ** $p < .01$, *** $p < .001$

The Combined Infant Sample was comprised of infants from Studies 1 and 2. Home language environment measures reflect hourly rates with silent periods removed. Covariate Set #1 included: LENA recording duration, child age at time of recording, dummy variable for study affiliation (Study 1 or Study 2). Covariate Set #2 included: Child gender, time between maternal report of perceived stress and LENA recording, dummy variables for race and ethnicity, and mother age. Income-to-needs-ratios were log-transformed.

Table S2

Relations among SES, Stress, and LENA- Child Sample Robustness Checks

	Adult Word Count				Conversation Turn Count			
	Model 1		Model 2		Model 3		Model 4	
	β	*SE*	*β*	*SE*	*β*	*SE*	*β*	*SE*
Perceived Stress	0.00	0.12	0.01	0.10	0.03	0.12	0.05	0.11
Education	0.33+	0.19	0.34*	0.16	0.20	0.19	0.17	0.17
Income-to-Needs	-0.23	0.18	-0.22	0.15	-0.09	0.18	-0.03	0.15
Covariate Set #1	yes		yes		yes		yes	
Covariate Set #2	yes		yes		yes		yes	
FIML used?	no		yes		no		yes	
Observations	72		95		72		95	

Note. + $p < .10$, * $p < 0.05$, ** $p < .01$, *** $p < .001$

The Child Sample was comprised of children from Study 3. Home language environment measures reflect hourly rates with silent periods removed. Covariate Set #1 included: LENA recording duration, and child age at time of recording. Covariate Set #2 included: Child gender, time between maternal report of perceived stress and LENA recording, dummy variables for race and ethnicity, and mother age. Income-to-needs-ratios were log-transformed.

Table S3

Relations among Stress, SES, and LENA- Study 1 Robustness Checks

	Adult Word Count				Conversation Turn Count			
	Model 1		Model 2		Model 3		Model 4	
	β	*SE*	*β*	*SE*	*β*	*SE*	*β*	*SE*
Perceived Stress	0.00	0.12	0.02	0.13	0.02	0.13	0.04	0.13
Education	0.15	0.15	0.05	0.17	0.07	0.16	-0.05	0.18
Income-to-Needs	1.15*	0.46	0.94+	0.49	0.81	0.50	0.64	0.52
Covariate Set #1	yes		yes		yes		yes	
Covariate Set #2	no		yes		no		yes	
FIML used?	no		no		no		no	
Observations	61		61		61		61	

Note. + $p < .10$, * $p < 0.05$, ** $p < .01$, *** $p < .001$

Home language environment measures reflect hourly rates with silent periods removed. Covariate Set #1 included: LENA recording duration, child age at time of recording. Covariate Set #2 included: Child gender, time between maternal report of perceived stress and LENA recording, dummy variables for race and ethnicity, and mother age. Income-to-needs-ratios were log-transformed.

Table S4

Relations among SES, Stress, and LENA- Study 2 Robustness Checks

	Adult Word Count				Conversation Turn Count			
	Model 1		Model 2		Model 3		Model 4	
	β	*SE*	*β*	*SE*	*β*	*SE*	*β*	*SE*
Perceived Stress	0.03	0.14	0.01	0.14	0.05	0.13	0.04	0.13
Education	0.32*	0.15	0.20	0.19	0.31*	0.15	0.15	0.19
Income-to-Needs	0.09	0.12	0.10	0.13	0.10	0.12	0.08	0.12
Covariate Set #1	yes		yes		yes		yes	
Covariate Set #2	no		yes		no		yes	
FIML used?	no		no		no		no	
Observations	62		62		62		62	

Note. + $p < .10$, * $p < 0.05$, ** $p < .01$, *** $p < .001$

Home language environment measures reflect hourly rates with silent periods removed. Covariate Set #1 included: LENA recording duration, child age at time of recording. Covariate Set #2 included: Child gender, time between maternal report of perceived stress and LENA recording, dummy variables for race and ethnicity, and mother age. Income-to-needs-ratios were log-transformed.

Table S5

Relations among SES, Stress, and LENA- Combined Infant Sample, 5- to 7-month-olds only

	Adult Word Count				Conversation Turn Count			
	Model 1		Model 2		Model 3		Model 4	
	β	*SE*	*β*	*SE*	*β*	*SE*	*β*	*SE*
Perceived Stress	-0.05	0.17	-0.06	0.19	0.03	0.16	-0.04	0.17
Education	0.80**	0.22	0.76*	0.30	0.51*	0.21	0.56*	0.27
Income-to-Needs	-0.18	0.31	-0.27	0.34	-0.34	0.29	-0.49	0.30
Covariate Set #1	yes		yes		yes		yes	
Covariate Set #2	no		yes		no		yes	
FIML used?	no		no		no		no	
Observations	42		42		42		42	

Note. + $p < .10$, * $p < 0.05$, ** $p < .01$, *** $p < .001$

The Combined Infant Sample was comprised of infants from Studies 1 and 2. Only families with infants between the ages of 5- and 7-months were included in the analyses. Home language environment measures reflect hourly rates with silent periods removed. Covariate Set #1 included: LENA recording duration, child age at time of recording, dummy variable for study affiliation (Study 1 or Study 2). Covariate Set #2 included: Child gender, time between maternal report of perceived stress and LENA recording, dummy variables for race and ethnicity, and mother age. Income-to-needs-ratios were log-transformed.

Table S6

Relations among SES, Physiological Stress, and LENA- Study 1 and Study 3

	Study 1				Study 3			
	Adult Word Count				Conversation Turn Count			
	Model 1		Model 2		Model 3		Model 4	
	β	*SE*	*β*	*SE*	*β*	*SE*	*β*	*SE*
Hair Cortisol Concentration	-0.09	0.16	0.07	0.16	0.03	0.12	-0.01	0.14
Education	0.19	0.20	0.27	0.20	0.47*	0.18	0.24	0.20
Income-to-Needs	0.50	0.64	-0.03	0.64	0.09	0.19	0.17	0.21
Covariate Set #1	yes		yes		yes		yes	
Covariate Set #2	no		no		no		no	
FIML used?	no		no		no		no	
Observations	47		47		50		50	

Note. + $p < .10$, * $p < 0.05$, ** $p < .01$, *** $p < .001$

Hair cortisol concentration was used as an index of physiological stress. Home language environment measures reflect hourly rates with silent periods removed. Covariate Set #1 included: LENA recording duration, child age at time of recording. Covariate Set #2 included: Child gender, time between maternal report of perceived stress and LENA recording, dummy variables for race and ethnicity, and mother age. Income-to-needs ratios and hair cortisol concentration were log-transformed.

Table S7

Relations among SES, Stress Composite, and LENA- All Studies and Combined Infant Sample

	Adult Word Count								Conversation Turn Count							
	Study 1		Study 2		Combined Infant Sample		Child Sample		Study 1		Study 2		Combined Infant Sample		Child Sample	
	β	*SE*	*β*	*SE*	*β*	*SE*	*β*	*SE*	*β*	*SE*	*β*	*SE*	*β*	*SE*	*β*	*SE*
Stress Composite	-0.07	0.21	-0.09	0.21	-0.08	0.12	-0.02	0.16	-0.02	0.22	0.09	0.20	-0.02	0.12	0.01	0.16
Education	0.06	0.16	0.18	0.19	0.19+	0.10	0.32+	0.18	-0.03	0.17	0.16	0.19	0.13	0.10	0.16	0.19
Income-to-Needs	0.88+	0.51	0.11	0.13	0.11	0.10	-0.23	0.17	0.58	0.55	0.07	0.12	0.10	0.10	-0.06	0.18
Covariate Set #1	yes		yes		yes		yes		yes		yes		yes		yes	
Covariate Set #2	yes		yes		yes		yes		yes		yes		yes		yes	
FIML used?	no		no		yes		no		no		no		yes		no	
Observations	63		62		227		73		63		62		227		73	

Note. + $p < .10$, * $p < 0.05$, ** $p < .01$, *** $p < .001$

The Combined Infant Sample was comprised of infants from Studies 1 and 2. Home language environment measures reflect hourly rates with silent periods removed. Covariate Set #1 included: LENA recording duration, child age at time of recording, dummy variable for study affiliation (Study 1 or Study 2). Covariate Set #2 included: Child gender, time between maternal report of perceived stress and LENA recording, dummy variables for race and ethnicity, and mother age. Income-to-needs-ratios were log-transformed.
